# Supplementary material for: Assessing empathy in adults: A Malay language validation and measurement invariance of the Empathy Quotient (M-EQ)
Source: PLoS One. 2026 May 19;21(5):e0349335. doi: 10.1371/journal.pone.0349335 (PMC13186360; doi:10.1371/journal.pone.0349335)
Supplement: S1 File — (DOCX) [file pone.0349335.s001.docx]

**(EQ- English version)
Empathy Quotient (EQ)**

**How to fill out the questionnaire**

*Below are a list of statements. Please read each statement very carefully and rate how strongly you agree or disagree with it by circling your answer. There are no right or wrong answers, or trick questions.*

**IN ORDER FOR THE SCALE TO BE VALID, YOU MUST ANSWER EVERY QUESTION.**

| *Examples* E1. | I would be very upset if I couldn’t listen to music every day. | strongly  agree | slightly  agree | slightly  disagree | strongly  disagree |
| --- | --- | --- | --- | --- | --- |
| E2. | I prefer to speak to my friends on the phone rather than write letters to them. | strongly  agree | slightly  agree | slightly  disagree | strongly  disagree |
| E3. | I have no desire to travel to different parts of the world. | strongly  agree | slightly  agree | slightly  disagree | strongly  disagree |
| E4. | I prefer to read than to dance. | strongly  agree | slightly  agree | slightly  disagree | strongly  disagree |
|  |  |  |  |  |  |

**PLEASE ANSWER ALL QUESTIONS.**

| 1. | I can easily tell if someone else wants to enter a conversation. | strongly  agree | slightly  agree | slightly  disagree | strongly  disagree | |
| --- | --- | --- | --- | --- | --- | --- |
| 2. | I find it difficult to explain to others things that I understand easily, when they don't understand it first time. | strongly  agree | slightly  agree | slightly  disagree | strongly  disagree | |
| ~~3.~~ | ~~I really enjoy caring for other people.~~ | ~~strongly~~  ~~agree~~ | ~~slightly~~  ~~agree~~ | ~~slightly~~  ~~disagree~~ | ~~strongly~~  ~~disagree~~ | |
| ~~4.~~ | ~~I find it hard to know what to do in a social situation.~~ | ~~strongly~~  ~~agree~~ | ~~slightly~~  ~~agree~~ | ~~slightly~~  ~~disagree~~ | ~~strongly~~  ~~disagree~~ | |
| 5. | People often tell me that I went too far in driving my point home in a discussion. | strongly  agree | slightly  agree | slightly  disagree | strongly  disagree | |
| ~~6.~~ | ~~It doesn't bother me too much if I am late meeting a friend.~~ | ~~strongly~~  ~~agree~~ | ~~slightly~~  ~~agree~~ | ~~slightly~~  ~~disagree~~ | ~~strongly~~  ~~disagree~~ | |
| 7. | Friendships and relationships are just too difficult, so I tend not to bother with them. | strongly  agree | slightly  agree | slightly  disagree | strongly  disagree | |
| 8. | I often find it difficult to judge if something is rude or polite. | strongly  agree | slightly  agree | slightly  disagree | strongly  disagree | |
| ~~9.~~ | ~~In a conversation, I tend to focus on my own thoughts rather than on what my listener might be thinking.~~ | ~~strongly~~  ~~agree~~ | ~~slightly~~  ~~agree~~ | ~~slightly~~  ~~disagree~~ | ~~strongly~~  ~~disagree~~ | |
| ~~10.~~ | ~~When I was a child, I enjoyed cutting up worms to see what would happen.~~ | ~~strongly~~  ~~agree~~ | ~~slightly~~  ~~agree~~ | ~~slightly~~  ~~disagree~~ | ~~strongly~~  ~~disagree~~ | |
| 11. | I can pick up quickly if someone says one thing but means another. | strongly  agree | slightly  agree | slightly  disagree | strongly  disagree | |
| 12. | It is hard for me to see why some things upset people so much. | strongly  agree | slightly  agree | slightly  disagree | strongly  disagree | |
| 13. | I find it easy to put myself in somebody else's shoes. | strongly  agree | slightly  agree | slightly  disagree | strongly  disagree | |
| 14. | I am good at predicting how someone will feel. | strongly  agree | slightly  agree | slightly  disagree | strongly  disagree | |
|  |  |  |  |  |  | |
| 15. | I am quick to spot when someone in a group is feeling awkward or uncomfortable. | strongly  agree | slightly  agree | slightly  disagree | strongly  disagree | |
| 16. | If I say something that someone else is offended by, I think that that's their problem, not mine. | strongly  agree | slightly  agree | slightly  disagree | strongly  disagree | |
| ~~17.~~ | ~~If anyone asked me if I liked their haircut, I would reply truthfully, even if I didn't like it.~~ | ~~strongly~~  ~~agree~~ | ~~slightly~~  ~~agree~~ | ~~slightly~~  ~~disagree~~ | ~~strongly~~  ~~disagree~~ | |
| 18. | I can't always see why someone should have felt offended by a remark. | strongly  agree | slightly  agree | slightly  disagree | strongly  disagree | |
| 19. | Seeing people cry doesn't really upset me. | strongly  agree | slightly  agree | slightly  disagree | strongly  disagree | |
| 20. | I am very blunt, which some people take to be rudeness, even though this is unintentional. | strongly  agree | slightly  agree | slightly  disagree | strongly  disagree | |
| 21. | I don’t tend to find social situations confusing. | strongly  agree | slightly  agree | slightly  disagree | strongly  disagree | |
|  |  |  |  |  |  | |
| 22. | Other people tell me I am good at understanding how they are feeling and what they are thinking. | strongly  agree | slightly  agree | slightly  disagree | strongly  disagree | |
| ~~23.~~ | ~~When I talk to people, I tend to talk about their experiences rather than my own.~~ | ~~strongly~~  ~~agree~~ | ~~slightly~~  ~~agree~~ | ~~slightly~~  ~~disagree~~ | ~~strongly~~  ~~disagree~~ | |
| ~~24.~~ | ~~It upsets me to see an animal in pain.~~ | ~~strongly~~  ~~agree~~ | ~~slightly~~  ~~agree~~ | ~~slightly~~  ~~disagree~~ | ~~strongly~~  ~~disagree~~ | |
| ~~25.~~ | ~~I am able to make decisions without being influenced by people's feelings.~~ | ~~strongly~~  ~~agree~~ | ~~slightly~~  ~~agree~~ | ~~slightly~~  ~~disagree~~ | ~~strongly~~  ~~disagree~~ | |
| 26. | I can easily tell if someone else is interested or bored with what I am saying. | strongly  agree | slightly  agree | slightly  disagree | strongly  disagree | |
| ~~27.~~ | ~~I get upset if I see people suffering on news programmes.~~ | ~~strongly~~  ~~agree~~ | ~~slightly~~  ~~agree~~ | ~~slightly~~  ~~disagree~~ | ~~strongly~~  ~~disagree~~ | |
| 28. | Friends usually talk to me about their problems as they say that I am very understanding. | strongly  agree | slightly  agree | slightly  disagree | strongly  disagree | |
| 29. | I can sense if I am intruding, even if the other person doesn't tell me. | strongly  agree | slightly  agree | slightly  disagree | strongly  disagree | |
|  |  |  |  |  |  |  |
| 30. | People sometimes tell me that I have gone too far with teasing. | strongly  agree | slightly  agree | slightly  disagree | strongly  disagree |  |
| 31. | Other people often say that I am insensitive, though I don’t always see why. | strongly  agree | slightly  agree | slightly  disagree | strongly  disagree |  |
| ~~32.~~ | ~~If I see a stranger in a group, I think that it is up to them to make an effort to join in.~~ | ~~strongly~~  ~~agree~~ | ~~slightly~~  ~~agree~~ | ~~slightly~~  ~~disagree~~ | ~~strongly~~  ~~disagree~~ |  |
| ~~33.~~ | ~~I usually stay emotionally detached when watching a film.~~ | ~~strongly~~  ~~agree~~ | ~~slightly~~  ~~agree~~ | ~~slightly~~  ~~disagree~~ | ~~strongly~~  ~~disagree~~ |  |
| 34. | I can tune into how someone else feels rapidly and intuitively. | strongly  agree | slightly  agree | slightly  disagree | strongly  disagree |  |
| 35. | I can easily work out what another person might want to talk about. | strongly  agree | slightly  agree | slightly  disagree | strongly  disagree |  |
| 36. | I can tell if someone is masking their true emotion. | strongly  agree | slightly  agree | slightly  disagree | strongly  disagree |  |
| ~~37.~~ | ~~I don't consciously work out the rules of social situations.~~ | ~~strongly~~  ~~agree~~ | ~~slightly~~  ~~agree~~ | ~~slightly~~  ~~disagree~~ | ~~strongly~~  ~~disagree~~ |  |
| 38. | I am good at predicting what someone will do. | strongly  agree | slightly  agree | slightly  disagree | strongly  disagree |  |
| ~~39.~~ | ~~I tend to get emotionally involved with a friend's problems.~~ | ~~strongly~~  ~~agree~~ | ~~slightly~~  ~~agree~~ | ~~slightly~~  ~~disagree~~ | ~~strongly~~  ~~disagree~~ |  |
| ~~40.~~ | ~~I can usually appreciate the other person's viewpoint, even if I don't agree with it.~~ | ~~strongly~~  ~~agree~~ | ~~slightly~~  ~~agree~~ | ~~slightly~~  ~~disagree~~ | ~~strongly~~  ~~disagree~~ |  |

Items with a strikethrough should not be used in the M-EQ.

For the M-EQ 23-item, please see <https://osf.io/c872d/files/t9nyp>
