# Supplementary material for: Assessing empathy in adults: A Malay language validation and measurement invariance of the Empathy Quotient (M-EQ)
Source: PLoS One. 2026 May 19;21(5):e0349335. doi: 10.1371/journal.pone.0349335 (PMC13186360; doi:10.1371/journal.pone.0349335)
Supplement: Attachment — Submitted filename: PlosOne response to the reviewers_7 Nov.docx [file pone.0349335.s003.docx]

7 November 2025

Dear Editor and Reviewers,

Thank you for this feedback and to help improve on this manuscript. Please see our responses below. All page numbers and line numbers are based on the clean version. Thank you.

Best wishes,

Min

**Reviewer #1:** 
Dear authors,
Thank you for your good manuscript, entitled "Assessing empathy in adults: A Malay language validation and measurement invariance of the Empathy Quotient (M-EQ)".

This is a valuable study. Here are my comments.
1. Title should change to “Empathy: Measurement invariance of the Empathy Quotient among Malaysian adults”.

*** We thank the reviewer for this suggestion. However, we would like to retain the existing title and not drop the ‘validation’ in the title because we had a 1-factor vs 3-factor structure analysed.

2. Keywords should change to “Empathy, Empathy Quotient (EQ), Measurement invariance, Psychometrics; Malaysia”.

*** We agree to drop two keywords; language type and gender. However we do not think it is appropriate to include ‘Malaysia’. The main interest is the Malay language and the Malay language is used in the Southeast Asia region, which meant that the M-EQ could be used in any of these countries.

3. Authors should add research questions/assumptions of their study in the end of Introduction section.

*** We thank the reviewer for this suggestion. We have now included the research question and hypotheses under Research Aim (see below and lines 91 to 99, and 118 to 119).

# Research aim

At present, there is no Malay version of the EQ. The Malay language is the national language in Malaysia, Indonesia, Brunei, and Singapore with approximately 220 million speakers. It is also widely spoken in southern Thailand, Philippines, and East Timor [21]. For this study, our aim was to develop and validate a Malay language version of the EQ (M-EQ). Based on the inconsistent factor models reported in previous reviews (see Table 1 and [5]), our first objective was to examine whether the M-EQ aligns better with a 1-factor or a 3-factor structure model. Our second objective was to assess its temporal reliability, and thus we conducted a test-retest over a 4-week interval. Additionally, our third objective was to evaluate measurement invariance (MI) on two variables; language types and gender.

…affective items. Therefore, our fourth objective was to examine whether M-EQ measures empathy equally across both genders.

4. Authors should write all items for the EQ in the Table (All statements).

*** We seek further clarity about this. The M-EQ is located at the end of this manuscript if the reviewer is asking about this. We have included footnotes for the abbreviations in each table as well.

5. Authors should write clinical implications of their study.

*** We thank the reviewer for this suggestion. We have now added this into the manuscript. See line 398 to 413.

Although our study focused on neurotypical adult population, we wish to highlight the potential application of the M-EQ in clinical settings. Some proposed that the multifaceted concept of empathy could contribute to a more complete understanding of various clinical populations such as autism (Robinson, 2019). In their study, they used EQ alongside Autism Spectrum Quotient (AQ) and the Relatives Questionnaire (RQ) (retrospective, parent/informant-report version of the Childhood Autism Spectrum Test) to facilitate quicker identification of autistic individuals and reduce diagnostic wait times. Another study reported that people with low emotional empathy was associated with Dark Triad traits, while low cognitive empathy are specifically linked to narcissism and Machiavellianism (Turner et al., 2019), indictaing EQ’s potential utility for psychopathy assessments. Scoring low on cognitive empathy factor is also linked to poor social function in schizophrenia patients (Michaels et al., 2014). Note that in both studies (Michaels et al., 2014; Turner et al., 2019), the authors focused on specific factors (cognitive or emotional empathy) in the EQ, suggesting that clinicians could streamline assessments by selecting only the most relevant items for their target population without compromising diagnostic validity. These studies underscore the potential for the M-EQ to be adapted for the clinical population in Malaysia, offering a culturally relevant and time-efficient tool for psychological assessment.

6. Authors should write doi for all references.
*** We apologise for omitting the DOIs and have now included them in the Reference List.

**Reviewer #2:** I appreciate the authors’ effort to validate the Malay version of the Empathy Quotient (M-EQ) and to examine its measurement invariance across gender and language type. However, the manuscript is not yet easy to follow, and several conceptual, methodological, and reporting issues currently limit its scientific contribution and clarity.

1. The manuscript lacks conceptual coherence. The introduction emphasizes cognitive and affective empathy, but the final model includes three factors (cognitive, emotional, and social skills) without adequate justification for the additional factor. Terminology is also inconsistent (e.g., “emotional” vs. “affective” empathy; “sex” vs. “gender”), which reduces clarity. The rationale for including “social skills” as a separate factor and its theoretical grounding should be explained more thoroughly.

*** We thank the reviewer for this. We have now included further explanation on the 3-factor structure as well as more explanation on the “social skills” in both the Introduction and Discussion sections. We also reviewed the entire manuscript by removing ‘sex’ and replaced it with ‘gender’.

2. It is unclear how Table 1 was compiled. Did the authors conduct their own review, or was the table adapted from previous work? The methodology for obtaining the data in Table 1 should be clarified. In addition, the table would be more useful if it included information about the populations studied (e.g., clinical vs. general population), age ranges, and other relevant demographics.

*** We have added more details to Table 1 to include demographic details such as mean and SD for age in the whole sample. We checked the details and realised that we omitted two studies (Gouveia et al., 2012; Paolo Senese et al., 2018) and removed one study (Dimitrijević et al., 2012) because they tested adolescents as young as 15 years of age. We excluded Dimitrijevic et al. (2012) because empathy in adolescent is still developing rapidly due to individual emotional experience and regulatory processes as well as biological differences between boys and girls (Gaspar & Esteves, 2022).

3. The manuscript does not adequately justify the use of two samples. It is unclear why the authors treated the datasets as separate for some analyses and combined them for others (e.g., measurement invariance). In addition, because data collection was interrupted by COVID-19, the comparability of pre- and post-pandemic data should be examined. The authors should report whether participants recruited before versus after the pandemic differed on key demographic or psychometric variables, as such differences could affect the factor structure and reliability estimates.

*** We apologise for this confusion. Data was collected from two different samples at two distinct time points (2019 and 2022). We collected one round each for Sample 1 and Sample 2 participants. Note that Sample 2 participants completed the M-EQ twice after a four-week interval.

We had two separate data collection for methodological rigour. This is because Sample 1 (n = 232) was tested against the original 1-factor structure using confirmatory factor analysis (CFA). Our results showed that the one-factor model was a poor fit and therefore we performed exploratory factor analysis (EFA). Results showed that a 3-factor model was a better fit. To avoid capitalizing on chance and adhering to best practices, we did not confirm the new factor structure within the same dataset. Instead, we collected new data (Sample 2, n = 354) to conduct a CFA, which confirmed the 3-factor model. We would like to iterate that the factor structure was replicated consistently across both samples, which strengthens confidence in the robustness of the 3-factor solution despite the different collection periods. We have now made this cleared under Analytical Plan to describe our approach to the two data collection. Please refer to line 167 to 171.

We implemented a 3-step validation process. In Step 1, we tested a one-factor model using confirmatory factor analysis (CFA) with AMOS 27 in Sample 1 participants. We then conducted an exploratory factor analysis (EFA) using SPSS 26 to better understand the underlying structure (Step 2). Based on the EFA results, a three-factor model was proposed to which we tested this using CFA on Sample 2 participants to confirm its validity (Step 3).

We appreciate the comment regarding pre- and post-pandemic but this is not feasible due to our data collection phase. Nonetheless, we have addressed the influence of COVID-19 on our participants and have acknowledged this in the Limitations section. See line 390 to 397.

…we acknowledge that our data collection was impacted by the COVID-19 pandemic. Some researchers reported that although cognitive and emotional empathy remained intact during the pandemic, there were significant reduction in empathic social skills (Baiano et al., 2022; Ng et al., 2024), suggesting that interventions targeting social skills would be ideal in helping others cope with mental health challenges. Other studies reported that those who maintained physical distancing and wearing of masks were prosocial indicators which helps to minimise spreading the COVID-19 virus (Pfattheicher et al., 2020). Taken together, these studies suggest empathic response could possibly be atypical during the pandemic outbreak and may have continued long after as a new ‘norm’.

4. The analytical strategy is not clearly explained and would benefit from stronger justification. The authors report testing only one- and three-factor models via CFA, yet EFA results are presented without any description of the procedure in the methods. In my opinion, a more rigorous approach would be to conduct EFA in Sample 1 to determine the optimal number of factors, followed by CFA in Sample 2 to confirm the structure. In addition, the CFA models tested show suboptimal fit indices (e.g., CFI < .900). I am therefore not convinced that the final model adequately fits the data. Relatedly, the criteria for model evaluation, construct validity testing, and measurement invariance thresholds should be more clearly justified and consistently applied.

*** We agree with the suggestion raised by the reviewer and did conduct these analyses. We apologise for the lack of clarity. We have addressed this comment in the above question as well.

5. Key results are underreported or presented unclearly. For example, chi-square statistics, degrees of freedom (rather than chi-square/df), and chi-square difference tests for invariance are not reported. RMSEA confidence intervals are missing despite being listed as evaluation criteria. Figures are difficult to interpret. Together, these issues make it difficult to evaluate the robustness of the findings.

*** We apologise for omitting these details. Please see revised on line 247 to 265 and Table 4.

6. Several psychometric concerns are also not adequately addressed.

(a) The Social Skills factor shows weak internal consistency (alpha = .619).

*** We agree with the reviewer regarding the low internal reliability in the SS factor and have now expanded this in the Discussion. See line 296 to 312.

…We acknowledge that the Social Skills (SS) Cronbach alpha raises questionable internal consistency (0.619) but note that this factor in the M-EQ only has four items and it is known that scales with fewer items tend to have lower alpha values to reduced item covariance. In the SS factor, the largest factor loading was for item 27 “I get upset if I see people suffering on news programmes“ with a 0.740 while the lowest was item 24 “It upsets me to see animals in pain“ at 0.342. Item 29 “I can sense if I am intruding, even if the other person doesn’t tell me“ and item 40 “I can usually appreciate the other person’s viewpoint, even if I don’t agree with it“ had moderate loadings at 0.367 and at 0.419 respectively. Although the reliability is lower than ideal, similar findings were reported in other studies [9,11,16]. For example, the Turkish EQ version removed 11 items including items 27 and 29 because the factor loadings were below 0.30 and challenges in translating these items into local cultural context [6,12]. Others reported that EQ is positively correlated to social desirability scores because empathizing does require individuals to be compliant with expectations from others [10,12,16]. Taken together, researchers need to be aware of interpreting SS factor on its own with caution. Nonetheless, given the theoretical relevance of the SS factor and its contribution to the overall empathy construct, we retained this factor in the M-EQ.

(b) Test-retest reliability values (r = .33-.62) are substantially lower than those reported in prior studies (Table 1) and warrant careful discussion.

*** Regarding the test-retest reliability value, we apologise for this mistake. After checking our data, our M-EQ test-retest from Sample 2 is *r* = .794, *p* < .001. See line 278.

… correlation coefficients (0.794, *p <* 0.001). This indicated that the 32 items of the M-EQ have...

(c) Partial scalar invariance across gender and language is acknowledged but not explained (e.g., why specific items function differently across subgroups).

*** We have now expanded the MI results on the partial scalar invariance for both gender and language types. Please see revised on line 335 to 348.

… differently. For example, in the CE factor, Item 30 “People sometimes tell me that I have gone too far with teasing” suggests that males and females may have different baseline tendencies on how teasing is perceived. One study showed that boys initiated teasing significantly more often than girls – 78% versus 22% of the time [33], suggesting that teasing may be a social norm common among males. However, teasing can also be interpreted as a form of aggression. Studies have shown that girls display more relational aggression while boys display more physical and verbal aggression [34] but this differences tend to converge in adulthood, indicating that teasing works equally across both genders later on in life [35]. Similarly, the intercept invariance observed for Item 11 “I can pick up quickly if someone says one thing but means another” in EE could be explained by early gendered socialisation. Girls are typically more verbal with their friends while boys engage in more physical play and roughhousing [36]. This difference may give females an advantage in detecting subtle cues in conversations, potentially influencing how they respond to this item.

… types (Kaplan, 1966). It is also possible this item is not an indicator of empathy. The term “offended” (translated as *kecil hati* in Malay) carries varying emotional weight or social implications to the speaker depending on cultural context. Rather than reflecting empathic concern, responses may be shaped by individual personality traits or cultural norms around emotional expression. Additionally, the reference to a vague ‘someone’ is ambiguous and requires participants to subjectively assess the emotional significance of the remark.

(d) The reduction from 40 to 32 items may be acceptable given low factor loadings, but the implications for comparability with the original EQ should be discussed in more depth.

*** We have added this to Discussion. See lines 313 to 323.

… Further, the M-EQ should be used as per the original EQ [3] with similar Likert scales scoring to obtain a total empathy score. Compared to the original EQ instrument, the M-EQ has 32 items across a 3-factor model; the CE factor has 15 items, EE factor 13 items and SS four items. Lawrence et al. [4] had 11 items in the CE and EE respectively with 6 for SS and the Korean version also had similar 28 items [9], suggesting that the M-EQ is concordant to past studies. Additionally, our item-level correlations showed good internal consistency in that most inter-item correlations were between 0.20 and 0.40. However, there were some items with weaker correlations e.g. item 6 in CE and item 37 in EE but these items also had lower factor loadings (0.358 and 0.419) respectively compared to the other items in each factor, suggesting that these items could be less central to their constructs. Overall, the M-EQ demonstrates structural and psychometric comparability to the original EQ.

7. It is unclear how the total scores were calculated (e.g., in the Sex Differences in Empathy section). Did the authors use factor scores or simply sum the items? If unweighted scores were used, the Social Skills factor, with only four items, would have very little influence on the total score. The authors should clarify their approach and discuss the implications for interpreting both the overall M-EQ score and the three subscale scores. They should also address how the M-EQ scale is intended to be used: should it be treated as three separate constructs (cognitive, emotional, and social skills) or as a single construct (empathy)? Depending on how the authors define the measure, it may be necessary to examine a higher-order CFA model (one second-order factor representing empathy, with three first-order factors representing cognitive, emotional, and social skills).

***We simply added the scores to obtain the total scores, and using the items identified in the EFA for the corresponding factor (refer to Table 3). There were 15 items in CE, 13 items in EE and four items in SS. We apologise for omitting on how to use the M-EQ. The M-EQ should be used similarly to the original EQ with similar Likert scales and scoring to obtain a total empathy score. Our three-factor model showed a good structure, and so we are confident if clinicians/practitioners wish to only select items from the CC and EE as previously reported in other studies (Michaels et al., 2014; Turner et al., 2019). We do not recommend the SS as a standalone due to sub-optimal reliability and that these items require further consideration pertaining to the individuals’ social perceptions and local cultural contexts. We have addressed these in the above question.

8. Finally, the authors should provide the data used for analysis. If full data sharing is not possible, they should include sufficient information (e.g., item-level correlations, means, and standard deviations) in the supplemental materials.

*** These data are now available in the supplementary.
