# Supplementary material for: Assessing empathy in adults: A Malay language validation and measurement invariance of the Empathy Quotient (M-EQ)
Source: PLoS One. 2026 May 19;21(5):e0349335. doi: 10.1371/journal.pone.0349335 (PMC13186360; doi:10.1371/journal.pone.0349335)
Supplement: Attachment — Submitted filename: PlosOne response to the reviewers_Feb 2026.docx [file pone.0349335.s004.docx]

19 February, 2026

Dear Editor and Reviewer,

Thank you for this feedback and it was much appreciated. Please see our responses below. All page numbers and line numbers are based on the clean version.

We have adopted the reviewer’s suggestions on changing our analytical approach and have now changed the almost the entire Results and Discussion sections to better reflect our findings.

Thank you.

Best wishes,

Min

***

Reviewer #2: I appreciate the authors’ efforts in revising the manuscript. Overall, the authors have addressed many of my previous concerns (as Reviewer 2). However, based on the dataset and AMOS outputs provided on the OSF platform (https://osf.io/c872d/files/osfstorage), as well as my own examination of the data, I identified one major issue related to the statistical analyses that requires serious attention.

Specifically, the reported models appear to have been substantially modified, likely on modification indices. Such extensive post hoc model modification has long been recognized as risking capitalization on chance and may lead to incorrect or unstable models (MacCallum et al., 1992). I am therefore strongly concerned about this practice. Although post hoc model modification is not inherently inappropriate, each added path must be theoretically justified and clearly documented.

As shown in Table 4, the configural model has 828 degrees of freedom, whereas the baseline configural model without additional paths (e.g., correlated residuals and cross-loadings) should have 922 degrees of freedom. This discrepancy (922-828 = 94) indicates that 94 additional parameters (or paths) were introduced to improve model fit without being explicitly reported to readers, although they are visible in the AMOS files. I might be persuaded that such modifications are justified if modification indices from both datasets consistently indicated the need for the same additional paths. However, the AMOS outputs do not support this claim. Moreover, the models include implausible paths, such as relations between a latent factor and an item residual. Taken together, these issues lead me to conclude that the reported models are unacceptable.

Instead of adding numerous post hoc paths, I think it may be preferable to remove poorly performing items (provided this does not compromise validity). For example, the authors may select the best few items per factor and conduct a CFA without correlated residuals or cross-loadings, or include such parameters only when they are theoretically justified.

In addition, there are numerous statistical issues throughout the manuscript. I strongly recommend that the authors carefully re-examine their analytic approach and results, or consult an expert in factor analysis or structural equation modeling. The following issues are those I was able to identify:

- Lines 178-179 (tracked-changes version): “Modification index coefficients were used to examine cross-loadings between items.” Please clarify whether correlated residuals were also examined. In addition, cross-loadings reflect relationships between items and multiple factors, not relationships between items themselves.

- The current standard for factor retention is parallel analysis, rather than relying solely on eigenvalues greater than 1. Please report the results of a parallel analysis.

- Please re-evaluate whether principal axis factoring (PAF) is appropriate given that the data were reported as normally distributed, or whether maximum likelihood (ML) estimation would be more suitable.

**Response:** Thank you for your feedback. We acknowledge that the statistical approach adopted in the previous version had limitations, particularly with factor retention and model specification. In response, we re-analysed the data following established best-practice recommendations for scale development and validation.

Please see page 13, line 161 to 192 under Analytical Approach.

Please see page 15, line 202 to page 20, line 265 under Factor Analysis. See below.

…We conducted EFA in sample 1 (n=232) in R. EFA was performed using maximum likelihood with oblimin rotation, given the expected correlations among factors. The data were suitable for factor analysis with KMO = 0.812, Bartlett p < .001. Factor retention was guided using Parallel Analysis. We used the following criteria: items were retained if they showed factor loading ≥ .40. Items were considered for removal when the items show cross-loading ≥ .30 on multiple factors and low communality (h² )< .20–.30 [30,31].

Parallel analysis initially supported a four-factor solution with an acceptable overall model quality (RMSEA= 0.037, RMSR = 0.05, TLI = 0.87). However, closer inspection of item‑level diagnostics indicated that this solution lacked psychometric and conceptual coherence. Several items have low communalities and some items have high cross-loading across factors indicating poor conceptual and psychometric coherence. Specifically, Factor 3 was defined by a single item (EQ27) with strong loading (λ = 0.82, h² = 0.68) while other items (EQ29, EQ40, and EQ39) showed weak loadings (0.32-0.36) and cross-loaded on Factor 4. A latent factor defined by only one item is considered psychometrically inadequate and was therefore deemed unacceptable. Similarly, items loading on factor 4 (EQ21, EQ22, EQ39 and EQ 25) showed weak factor loading (0.39 to 0.37 with cross loading), thus undermining this factor. Taken together, these findings suggest that the 4-factor solution represented an over extraction of factors and thus rejected.

We then removed seven items (EQ2, EQ3, EQ4, EQ17, EQ23, EQ24, and EQ25) for consistently low loadings across factors. EFA was re-estimated and parallel analysis was conducted again. Findings indicated a 3-factor solution with improved overall model quality (RMSEA= 0.041, RMSR= 0.05, TLI= 0.88). However, factor 3 remained psychometrically invalid with only EQ27 (factor loading of 1.00), indicating statistical over-extraction.

Given these findings, we evaluated a 2-factor solution and removed item EQ27 which was supported by parallel analysis. Model fit indices remained acceptable (RMSEA= 0.042, RMSR= 0.06, TLI= 0.87), and both factors were statistically acceptable. Factor 1 comprised of 13 items (EQ1, EQ11, EQ13, EQ14, EQ15, EQ22, EQ26, EQ28, EQ29, EQ34, EQ35, EQ36, and EQ38) with factor loadings ranging from 0.409-0.701. These 13 items were about the sharing an emotional state, a willingness to help others, and feeling distress or upset to another’s pain. An example was “I can pick up quickly if someone says one thing but means another – Item 11”. In sum, this factor was conceptualised as “emotional empathy” (EE). Factor 2 also comprised of 13 items (EQ5, EQ7, EQ8, EQ10, EQ12, EQ16, EQ18, EQ19, EQ20, EQ30, EQ31, EQ32, and EQ33) with factor loadings ranging from 0.411 to 0.580. These items contain perspective-taking, understand other’s people feelings, and imagined what was others’ perspectives. Example of an item is “I often find it difficult to judge if something is rude or polite – item 8”. Hence, this factor was conceptualised as “cognitive empathy” (CE).

Table 3. Factor loadings for M-EQ from EFA using the oblimin method (n = 232)

To confirm the structural validity of M-EQ with 26 items, we repeated CFA for 2-factor model with Sample 2 participants (n = 354) using weighted least squares mean‑ and variance‑adjusted (WLSMV) estimation. The model specification was informed by the EFA results, and 14 items previously identified as problematic were excluded prior to model estimation. Our results showed an adequate model of fit: χ²(298) = 671.96, p < .001; χ²/df = 2.25, CFI = 0.963, TLI = 0.960, RMSEA = 0.060 (90% CI .054–.066), SRMR = 0.074. Factor 1 (EE) was very strong, with all items loading above .50. Factor 2 (CE) showed acceptable fit, but three items (EQ10, 32, 33) exhibited very weak standardized loadings (< .30), indicating they did not reliably measure the latent construct in this sample. As such, these three items were removed and the CFA was re-estimated.

The refined two-factor model (23 items) demonstrated good fit to the data, χ²(229) = 528.40, p < .001; χ²/df = 2.31; CFI = .970; TLI = .967; RMSEA = .061 (90% CI [.054, .068]); SRMR = .072. Standardized factor loadings ranged from 0.51 to 0.77 for Factor 1 (EE) and from 0.35 to 0.76 for Factor 2 (CE). No post-hoc model modifications were applied. Although EQ19 showed a relatively lower standardized loading (0.353), it exceeded the minimum acceptable threshold and was retained to preserve content coverage of Factor 2. In addition, removing EQ19 did not result in a meaningful improvement in overall model fit.

We next estimated a second-order CFA model in which EE and CE factors were loaded onto a higher-order empathy construct using R (Lavaan package). The model did not converge to a proper solution, and produced a non‑invertible information matrix and a negative variance estimate for the second-order factor, indicating that the data does not support a unidimensional structure. Therefore, the correlated 2-factor model was retained for as the appropriate structural representation of the M-EQ, χ²(229) = 481.206, CFI = 0.973, TLI = 0.971, RMSEA = 0.056, SRMR = 0.070, with the latent factors showing a moderate negative correlation (r = −0.434, p < .001)….

***

“Cattell’s scree plot” should be spelled with a double t (not “Catell”).

**Response:** This is no longer applicable.

***

Please provide a citation for the measurement invariance criteria (ΔCFI > -0.01, ΔRMSEA < 0.01, and ΔSRMR < 0.01).

**Response:** This is no longer applicable as we have now changed the MI analytical approach to Item Response Theory-based Differential Item Functioning (DIF) analyses. Please see the revised section.

Please see page 20, line 267 to 288.

**Measurement Invariance (MI) for M-EQ**

Gender. Measurement invariance across gender was first examined using single‑group CFAs. Model fit was poor for males (CFI = 0.77) and inadequate for females (CFI = 0.86), indicating that configural invariance could not be established and thus multi-group CFA invariance testing was not conducted. Instead, gender-related measurement equivalence was examined using Item Response Theory-based Differential Item Functioning (DIF) analyses. First, we examined a posteriori (EAP) reliability for both subscales and it was high for EE (0.865) and acceptable for CE (0.775), supporting the use of latent scores for group comparisons. DIF analyses revealed no evidence of gender-related DIF for either EE or CE. Likelihood-ratio DIF tests were conducted for discrimination (a1) and threshold (d1–d3) parameters, with p-values adjusted using the Benjamini–Hochberg false discovery rate procedure. No items showed statistically significant DIF and none of the drop‑in‑fit tests indicated meaningful improvements in model fit. These indicate invariant item functioning across gender groups (see Table 4).

Language types. We repeated the analyses using the sample 2 (Latin: English/Malay = 234, non-Latin: Chinese/Tamil = 120). EAP reliability estimates again indicated adequate measurement precision for latent mean comparisons (EE = 0.862; CE = 0.767). DIF analyses showed no evidence of differential item functioning for either EE or CE across language groups. Likelihood-ratio DIF tests for discrimination (a1) and threshold (d1–d3) parameters, with Benjamini–Hochberg FDR correction, yielded no significant item-level differences (all Δ−2LL = 0). These results indicate invariant item functioning across language types, suggesting that M -EQ items function equivalently for respondents using Latin and non-Latin as their first language (see Table 4).

***

Please clarify the following statement: “Should configural invariance not be achieved, partial invariance was tested” Why was partial invariance considered only at the configural level?

**Response:** This is no longer applicable as we have now changed the MI analytical approach. See above.

***

- Lines 214-215: “The correlation values between the three factors were weak to moderate (-0.39 to 3.98).” Correlation coefficients should fall between -1 and 1.

**Response:** We acknowledge this mistake. However, this is no longer applicable because we have now changed the approach as recommended by the reviewer.

***

- Lines 240-242: “χ²/df = 1.547, p < .001, CFI = 0.904, GFI = 0.895, AGFI = 0.846, TLI = 0.884, RMSEA = 0.039, SRMR = 0.055.” GFI and AGFI were not specified in the Analysis Plan section. Please also justify why model fit was deemed adequate when, for example, the TLI did not meet the stated cutoff. –

**Response:** This is no longer applicable because we have now changed the approach as recommended by the reviewer.

***

As noted in my previous review, please report the degrees of freedom for all tested models. Without df, it is not possible to evaluate the extent of model modification without access to the raw data.

**Response:** We apologize for omitting the degrees of freedom. We have now included this detail across the Results section.

** see page 19, line 196 - 198.

… Results showed an inadequate model of fit for 1-factor: χ² (740) = 1901.8, *p* < .001, χ²/df = 2.57, RMSEA = 0.082, SRMR = 0.1177; CFI = .449; TLI = .419. These …

** see page 18, line 245 - 246.

… results showed an adequate model of fit: χ²(298) = 671.96, p < .001; χ²/df = 2.25, CFI = 0.963, TLI = 0.960, RMSEA = 0.060 (90% CI .054–.066), SRMR = 0.074. Factor 1 (EE) is very strong…

** see page 18, line 259 - 253.

The refined two-factor model (23 items) demonstrated good fit to the data, χ²(229) = 528.40, p < .001; χ²/df = 2.31; CFI = .970; TLI = .967; RMSEA = .061 (90% CI [.054, .068]); SRMR = .072.

***

There should be multiple test-retest reliability values, one for the scale and one for each factor.

**Response:** We agree with the reviewer. We have now reported both reliability values for Time (Time 1 and Time 2), and scores (per subscale: EE, CE, and total M-EQ).

** see page 23, line 307 - 311.

… Test–retest reliability was examined using Pearson correlations between Time 1 and Time 2 scores. The EE subscale demonstrated good temporal stability (r = 0.734, p < .001), as did the CE subscale (r = 0.748, p < .001). The total empathy score showed moderate test–retest reliability (r = 0.805, p < .001). Overall, these findings indicate satisfactory temporal stability, particularly for the subscale scores.

***

Please clarify whether internal consistency estimates were examined separately for Sample 1 and Sample 2.

**Response:** We performed internal consistency for Sample 2 only using Cronbach’s alpha (α) and McDonald’s omega (ωₜ).

** see page 23, line 298 – 306.

Internal consistency of the M-EQ 23 items was evaluated using both Cronbach’s α and McDonald’s ωₜ. The EE subscale demonstrated good reliability (α = .86, ωₜ = .86), and the CE subscale showed acceptable reliability (α = .78, ωₜ = .78). As the scale exhibited a multidimensional structure, Cronbach’s alpha for the total score was lower (α = .67), which is expected for scales comprising multiple correlated dimensions, as alpha assumes unidimensionality and equivalent factor loadings. When these assumptions are violated, alpha tends to underestimate reliability [32,33]. Therefore, McDonald’s omega was also computed for the total score, yielding high composite reliability (ωₜ = .88). Subscale scores are emphasized for interpretation, with the total score reported as a supplementary index.

***

Thank you for the clarification regarding the computation of total and subscale scores. However, given that the instrument is described as comprising three correlated first-order factors while also being used to generate a single total empathy score, it remains unclear whether the authors tested a second-order CFA model in which cognitive, emotional, and social skills factors load onto a single higher-order empathy construct. The unequal number of items across the three factors has important implications for the interpretation of the total score when unweighted sums are used. This issue requires explicit discussion in the manuscript. In sum, substantial revisions are still required before this manuscript can be considered suitable for publication. I hope these comments will be helpful to the authors.

**Response:** We thank the reviewer for raising the important point regarding the structural relationship between the subscales and the use of a total score. We acknowledge that when the reviewer first raised this, there was three factors identified which made logical sense to run second-order CFA.

However, in our revised analysis, the M-EQ now has 2 factors and 23 items: 13 items in Emotional Empathy (EE) and 10 items in Cognitive Empathy (CE) subscale. We did estimate a second‑order CFA in which the two first‑order factors identified in our EFA/CFA (Emotional Empathy and Cognitive Empathy) (see page 19, line 258 to 265) were specified to load onto a higher‑order empathy factor. The model was estimated in *R* using the lavaan package with WLSMV estimation.

When a second‑order model was imposed, the result showed a non‑invertible information matrix and negative latent variance. It provides no information beyond the correlation between the two factors. This suggests that the hierarchical model is not supported and more so since there were no other theoretical and statistical constraints imposed.

We retained the two-factor model and results showed that the correlated two‑factor model remained well‑fitting, χ²(229) = 481.206, CFI = 0.973, TLI = 0.971, RMSEA = 0.056, SRMR = 0.070, with a moderate correlation between Emotional and Cognitive Empathy (*r* = –.434, *p* < .001). This pattern indicates that while the factors are related, they do not form a unidimensional structure that would justify a higher‑order model.

For these reasons, we retained the correlated two‑factor model as the most appropriate representation of the data. Because the subscales differ in item length and represent distinct but related constructs, subscale scores are emphasized as the primary outcomes, with the total score reported only as a supplementary composite measure, whose reliability is supported by omega total (ωₜ = .88).
