# Supplementary material for: Assessing empathy in adults: A Malay language validation and measurement invariance of the Empathy Quotient (M-EQ)
Source: PLoS One. 2026 May 19;21(5):e0349335. doi: 10.1371/journal.pone.0349335 (PMC13186360; doi:10.1371/journal.pone.0349335)
Supplement: Attachment — Submitted filename: PlosOne response to the reviewers_R2_April.docx [file pone.0349335.s005.docx]

16 April, 2026

Dear Editor and Reviewer,

Thank you for this feedback and it was much appreciated. Please see our responses below. All page numbers and line numbers are based on the clean version.

We have adopted the reviewer’s suggestions on changing our analytical approach and have now changed the almost the entire Results and Discussion sections to better reflect our findings.

Thank you.

Best wishes,

Min

***

Additional Editor Comments:

- Please provide more details and substantial explanations and references of why Item Response Theory-based Differential Item Functioning (DIF) analyses were used instead of multi-group SEM (P. 29 and 34).

**Response:** Thank you for your feedback. This was raised by Reviewer 2 as well and our response is included under the Reviewer 2 below.

- Please discuss about the omitted items from the scale to demonstrate that no essential elements have been removed.

**Response:** From the original 40 items, we now have 23 items which meant that we omitted 17 items in total. While this number appears high, Muncer and Ling (2006) had 15 items in their revised version and this was supported by several others (Gouveia et al., 2012; Paolo Senese et al., 2018; Zhao et al., 2018).

As per our Discussion (see p. 24 - 26), the items identified as cognitive empathy, emotional empathy and social skills domains were different across languages. This suggests that interpretation of these items could be a result of the language. The variability across language versions suggests that certain items may be more sensitive to linguistic and cultural interpretation rather than core empathic capacity per se, supporting their removal in the interest of cross-cultural validity.

We believe that our robust analytical approach with the EFA, CFA implemented is sound psychometrically. The omitted items predominantly consisted of those with weak factor loadings, substantial cross-loadings across domains, or low communalities, indicating limited contribution to the latent constructs in the Malay language context. Further, the omitted items did not represent unique conceptual facets of empathy that were absent from the retained items; rather, they overlapped substantially in content with retained indicators within each domain. One clear difference between M-EQ and other studies is the absence of the Social Skills (SS) domain. The SS domain has often been reported with low internal consistency with some indicating that this domain is related to social desirability (Berthoz et al., 2008; Kim & Lee, 2010; Kose et al., 2018; Preti et al., 2011).

In addition to statistical criteria, the final item set was evaluated through expert review by two bilingual authors with subject-matter expertise, who independently confirmed that the retained items adequately capture the essential conceptual content of each empathy domain.

***

… other languages reported a 1-factor (Zhao et al., 2018), 4-factor (Zhang et al., 2018) but the majority showed a 3-factor model with EE, CE, and SS (Berthoz et al., 2008; Gouveia et al., 2012; Groen et al., 2015; Kim & Lee, 2010; Kose et al., 2018; Kosonogov, 2014; Paolo Senese et al., 2018; Preti et al., 2011). Yet some acknowledged issues pertaining to SS and EE. One study showed multiple loading on the same item across two factors (Kose et al., 2018) and another suspected that the SS factor is better suited to measure personality type i.e. assertiveness rather than an empathy dimension (Gouveia et al., 2012). Others reported lower consistency and…

… items in their analysis but others identified different items for CE, EE and SS respectively (Groen et al., 2015; Preti et al., 2011), indicating interpretation of these items may mean differently due to subtle nuances in the translated language, rather than core empathic capacity per se. Taken together, we are confident that our analyses for the 23-item M-EQ is robust psychometrically and very much aligned with theoretical underpinning empathy dimensions. Importantly, all core domains and subcomponents of the original EQ framework remain represented in the final item set, with each domain retaining multiple indicators reflecting its theoretical breadth. The omitted items predominantly consisted of those with weak factor loadings, substantial cross-loadings across domains, or low communalities, indicating limited contribution to the latent constructs in the Malay language context. Further, the omitted items did not represent unique conceptual facets of empathy that were absent from the retained items; rather, they overlapped substantially in content with retained indicators within each domain. As such, item removal reflects refinement of measurement rather than loss of conceptual content, ensuring preservation of essential empathic elements while improving clarity and cultural suitability….

- The first objective "our first objective was to examine whether the M-EQ aligns better with a 1-factor or a 3-factor structure model." should be revised to fit with the current finding

**Response:** We agree that we need to change this sentence, but we disagree in revising to fit the current finding. We have now changed this in Abstract, Introduction and Discussion to better reflect our position.

**Reviewer #2:**

I appreciate the substantial effort the authors have made in revising the manuscript. The paper is clearly improved, and the overall model-building strategy is now much clearer than in the previous version.

That said, my main concern for this revision is the new measurement invariance analysis. The results showed that the configural multigroup CFA did not fit well, thus measurement invariance was not established. From my understanding, however, the authors then shifted to IRT-based DIF analyses for the two subscales (EE, CE) separately, which changes the model being tested rather than resolving the original issue that the 2-factor model did not fit the data well across groups. I do not see a clear theoretical justification for analyzing the subscales separately. Therefore, I do not think the current evidence is sufficient to support a broad claim of measurement invariance.

Because the files on OSF do not allow full replication of the reported results, I can only infer what may have occurred. Typically, if a single-group CFA model fits the data excellently, the corresponding multigroup model should also show at least acceptable fit, which does not appear to be the case here. One possibility is that the single-group analyses used WLSMV, which may yield more favorable fit indices in some situations (Xia & Yang, 2019), whereas the multigroup analyses may have relied on maximum likelihood, resulting in substantially poorer fit. It is also important to note that the procedure for testing measurement invariance with WLSMV differs from that used in standard CFA with maximum likelihood (Sass, Schmitt, & Marsh, 2014). Thus, if the authors would like to return to the CFA framework for measurement invariance using the WLSMV estimator, they should follow established best practices. If the authors instead wish to use IRT, they should do so consistently and describe the technique more thoroughly so that readers can clearly understand the analytic approach.

**Response:** We thank the reviewer for the constructive feedback regarding the measurement invariance analyses. We agree that clarity and methodological consistency are essential in evaluating cross-group equivalence, and we have substantially revised this section of the manuscript accordingly.

First, we re-examined all measurement invariance analyses using a consistent CFA framework in R (*lavaan*), applying an estimator appropriate for ordinal data using WLSMV (see Methods – Analytical Plan p.15). In the previous version, the previous version included a mixture of WLSMV (single‑group) and ML-based estimation (multigroup), which may have artificially inflated differences in model fit. These inconsistencies have now been fully resolved. In summary, the revised analyses show acceptable single-group fit for gender, acceptable configural model fit across groups, and that there is support for metric invariance based on recommended ΔCFI and ΔRMSEA thresholds although there is some elevation in SRMR, which we then discussed this in the Discussion.

Second, we clarify that we it was not our intention to shift from CFA to IRT as an alternative modelling approach. In the revised manuscript, multigroup CFA remains the primary method to evaluate measurement invariance. The results now show that the configural model demonstrates acceptable overall fit, and metric invariance is supported based on established criteria (ΔCFI and ΔRMSEA) with some degree of model misfit remains (e.g., elevated SRMR values) (see Results on p.20 to 21). We also now provide a cautious interpretation, avoiding any broad claim of full invariance given the slightly elevated SRMR values in some groups.

To avoid confusion, we now clearly state that the IRT-based DIF analyses were conducted as supplementary item-level diagnostics rather than as a replacement for CFA-based invariance testing. The purpose was to examine whether the modest misfit in some groups (e.g. in non-Latin language group) could be attributed to specific items functioning differently. Importantly, the DIF analyses were conducted within each subscale (EE and CE) to align with the established two-factor structure, rather than to redefine the measurement model.

***

… Once the construct validity of the M-EQ is determined, we evaluated measurement invariance following a 2-stage analytic strategy grounded primarily in CFA. We conducted multi-group CFA to examine invariance across gender and language types using WLSMV estimator in R (*lavaan* package). Measurement invariance was assessed sequentially. First, we tested configural invariance to determine whether the two‑factor structure was comparable across groups. Next, we tested metric invariance by constraining factor loadings to equality. Model fit was evaluated using CFI, RMSEA, and SRMR, and changes in fit were judged using recommended criteria (ΔCFI ≤ .010, ΔRMSEA ≤ .015, ΔSRMR ≤ 0.030) (Chen, 2007). Given known sensitivity of SRMR in WLSMV models, this index was interpreted cautiously.

To supplement the CFA results and to explore potential item-level sources of misfit, we conducted item response theory–based differential item functioning (DIF) analyses. These analyses were intended as diagnostic tools to identify whether specific items functioned differently across groups. Separate multigroup graded response models were estimated for the EE and CE subscales to align with the established two‑factor structure. Anchor items were selected a priori and used to link groups to a common latent metric. DIF was evaluated using likelihood‑ratio tests comparing nested models, with Benjamini–Hochberg correction applied to control for false discovery. Expected a posteriori (EAP) reliability was computed to assess the precision of latent trait estimates.

…

**Gender.** Measurement invariance across gender was first examined using single‑group CFAs. Single-group CFA indicated acceptable fit in the female group (CFI = 0.975, TLI = 0.972, RMSEA = 0.061, SRMR = 0.080), and the male group also showed acceptable CFI, TLI, and RMSEA values but elevated SRMR (CFI = 0.944, TLI = 0.938, RMSEA = 0.067, SRMR = 0.103). We next proceed with the configural model, and it demonstrated acceptable overall fit across gender (CFI = 0.968, TLI = 0.965, RMSEA = 0.063, SRMR = 0.088), indicating that the two-factor structure was broadly similar across male and female participants. Constraining factor loadings to equality across gender resulted in acceptable model fit (CFI = 0.959, RMSEA = 0.070, SRMR = 0.093), with minimal changes in fit indices (ΔCFI = −0.009, ΔRMSEA = +0.007), supporting metric invariance. However, the slightly elevated SRMR values warrant cautious interpretation. We further examined gender-related item level equivalence using Item Response Theory-based Differential Item Functioning (DIF) analyses. First, we examined a posteriori…

**Language types.** We repeated the analyses using the sample 2 (Latin: English/Malay = 234, non-Latin: Chinese/Tamil = 120). Single-group CFA indicated acceptable fit for the Latin group (CFI = 0.964, RMSEA = 0.062, SRMR= 0.082), whereas the non-Latin group showed acceptable but slightly poorer fit for CFI particularly in terms of RMSEA and SRMR (CFI = 0.957, RMSEA = 0.089, SRMR = 0.108). The configural model demonstrated acceptable overall fit (CFI = 0.961, RMSEA = 0.072, SRMR = 0.091), indicating that the two-factor structure was broadly similar across language groups, although model fit was not optimal. Constraining factor loadings to equality resulted in minimal changes in model fit (metric model: CFI = 0.953, RMSEA = 0.077, SRMR = 0.096; ΔCFI = −0.008, ΔRMSEA = +0.005), suggesting support for metric invariance across language groups. However, given the elevated SRMR values and poorer fit in the non-Latin group, these findings should be interpreted with caution. DIF analyses were conducted to further examine potential item-level non-invariance. EAP reliability estimates again indicated adequate measurement precision for latent mean comparisons (EE = 0.862; CE = 0.767). DIF analyses showed no evidence of differential item…

---

Moreover, I encourage the authors to strengthen the limitations section. First, they should explicitly acknowledge the extent of model refinement that occurred during the revision process, including the iterative and partly data-driven nature of the final 2-factor solution. Second, the manuscript currently provides limited evidence of validity beyond factor analyses. The revised paper relies mainly on EFA, CFA, and IRT-based analyses, and even these provide only partial support for the revised measurement model. This should be stated more clearly as a limitation. In addition, the overall language of the manuscript should be softened so that the conclusions more accurately reflect the strength of the evidence. Relatedly, the authors should carefully review the abstract, methods, results, discussion, and tables for consistency, as the analytic strategy changed substantially and all sections should align with the revised model and its limitations. It may also be helpful to include figures illustrating the mesurement models.

**Response:** We agree with the reviewer. We have now revised Limitations section (see p.29 to 30).

… We acknowledge that there are several limitations in our study. First, the final two‑factor, 23‑item structure of the M‑EQ emerged through an iterative process that included both theory‑driven reasoning and data‑driven refinement. Although this approach is common in early-stage instrument validation, it raises the possibility that the model may partly reflect sample‑specific characteristics. Although we have a large sample, we acknowledge that our sampling was not sufficiently diverse to represent the human lifespan because our sample is entirely young adults. Further, our university student sampling limits the generalisability to the wider population. Another limitation is that we did not directly measure intelligence. While the EQ does require some verbal intelligence, 99.4% of our sample completed a minimum of 12 years of formal education, suggesting that they do have at least average intelligence.

Second, although the study provides initial evidence for construct validity based on EFA, CFA, and supplementary IRT‑based DIF analyses, the validity evidence is limited in scope because our evaluation focused primarily on the internal structure. We did not include other behavioural measures such as emotion recognition tasks (Besel & Yuille, 2010) or other empathy scales such as Toronto Empathy Questionnaire (Spreng et al., 2009) to assess convergent, discriminant or predictive validity. As such, conclusions regarding the construct validity of the M‑EQ should be considered preliminary and strengthened in future work using multi‑method validation approaches and across different samples. While the measurement invariance results support broad comparability of the M‑EQ across gender and language groups; the evidence is not unequivocal. While configural and metric invariance were supported based on recommended fit index changes, some single‑group and multigroup models showed elevated SRMR and RMSEA values, particularly in the non‑Latin language group. These patterns indicate localized model misfit, and full equivalence should not be assumed without caution.

Lastly, we acknowledge that our data collection was impacted by the COVID-19 pandemic. Some researchers reported that although cognitive and emotional empathy remained intact during the pandemic, there were significant reduction in empathic social skills (Baiano et al., 2022; Ng et al., 2024), indicating that empathic response could possibly be atypical during the pandemic outbreak and may have continued long after as a new ‘norm’….

---

In summary, although the manuscript is substantially improved, important concerns remain, particularly regarding the measurement invariance analysis. Addressing these issues may substantially change the study’s conclusions.

**Response:** We appreciate all enquiries and suggestions raised for this manuscript as it helped us on improving this manuscript.

**References**

Baiano, C., Raimo, G., Zappullo, I., Marra, M., Cecere, R., Trojano, L., & Conson, M. (2022). Empathy through the Pandemic: Changes of Different Emphatic Dimensions during the COVID-19 Outbreak. *International Journal of Environmental Research and Public Health*, *19*(4), Article 4. https://doi.org/10.3390/ijerph19042435

Berthoz, S., Wessa, M., Kedia, G., Wicker, B., & Grèzes, J. (2008). Cross-Cultural Validation of the Empathy Quotient in a French-Speaking Sample. *The Canadian Journal of Psychiatry*, *53*(7), 469–477. https://doi.org/10.1177/070674370805300712

Besel, L. D. S., & Yuille, J. C. (2010). Individual differences in empathy: The role of facial expression recognition. *Personality and Individual Differences*, *49*(2), 107–112. https://doi.org/10.1016/j.paid.2010.03.013

Chen, F. F. (2007). Sensitivity of Goodness of Fit Indexes to Lack of Measurement Invariance. *Structural Equation Modeling: A Multidisciplinary Journal*, *14*(3), 464–504. https://doi.org/10.1080/10705510701301834

Gouveia, V. V., Milfont, T. L., Gouveia, R. S. V., Neto, J. R., & Galvão, L. (2012). Brazilian-Portuguese Empathy Quotient: Evidences of its Construct Validity and Reliability. *The Spanish Journal of Psychology*, *15*(2), 777–782. https://doi.org/10.5209/rev_SJOP.2012.v15.n2.38889

Groen, Y., Fuermaier, A. B. M., Den Heijer, A. E., Tucha, O., & Althaus, M. (2015). The Empathy and Systemizing Quotient: The Psychometric Properties of the Dutch Version and a Review of the Cross-Cultural Stability. *Journal of Autism and Developmental Disorders*, *45*(9), 2848–2864. https://doi.org/10.1007/s10803-015-2448-z

Kim, J., & Lee, S. J. (2010). Reliability and Validity of the Korean Version of the Empathy Quotient Scale. *Psychiatry Investigation*, *7*(1), 24–30. https://doi.org/10.4306/pi.2010.7.1.24

Kose, S., Celikel, F. C., Kulacaoglu, F., Akin, E., Yalcin, M., & Ceylan, V. (2018). Reliability, validity, and factorial structure of the Turkish version of the Empathy Quotient (Turkish EQ). *Psychiatry and Clinical Psychopharmacology*, *28*(3), 300–305. https://doi.org/10.1080/24750573.2018.1462039

Kosonogov, V. (2014). The psychometric properties of the Russian version of the Empathy Quotient. *Psychology in Russia*, *7*(1), 96–104. https://doi.org/10.11621/pir.2014.0110

Muncer, S. J., & Ling, J. (2006). Psychometric analysis of the empathy quotient (EQ) scale. *Personality and Individual Differences*, *40*(6), 1111–1119. https://doi.org/10.1016/j.paid.2005.09.020

Ng, J. Y., Lin, M.-H., & Yong, M. H. (2024). Malaysians’ mental health and lifestyle behavior during the COVID-19 pandemic: A qualitative approach. *Academia Mental Health and Well-Being*, *1*(3). https://doi.org/doi.org/10.20935/MHealthWellB7375

Paolo Senese, V., De Nicola, A., Passaro, A., & Ruggiero, G. (2018). The Factorial Structure of a 15-Item Version of the Italian Empathy	Quotient Scale. *European Journal of Psychological Assessment*, *34*(5), 344–351. https://doi.org/10.1027/1015-5759/a000348

Preti, A., Vellante, M., Baron-Cohen, S., Zucca, G., Petretto, D. R., & Masala, C. (2011). The Empathy Quotient: A cross-cultural comparison of the Italian version. *Cognitive Neuropsychiatry*, *16*(1), 50–70. https://doi.org/10.1080/13546801003790982

Spreng, R. N., McKinnon, M. C., Mar, R. A., & Levine, B. (2009). The Toronto Empathy Questionnaire: Scale Development and Initial Validation of a Factor-Analytic Solution to Multiple Empathy Measures. *Journal of Personality Assessment*, *91*(1), 62–71. https://doi.org/10.1080/00223890802484381

Zhang, Y., Xiang, J., Wen, J., Bian, W., Sun, L., & Bai, Z. (2018). Psychometric properties of the Chinese version of the empathy quotient among Chinese minority college students. *Annals of General Psychiatry*, *17*(1), 38. https://doi.org/10.1186/s12991-018-0209-z

Zhao, Q., Neumann, D. L., Cao, X., Baron-Cohen, S., Sun, X., Cao, Y., Yan, C., Wang, Y., Shao, L., & Shum, D. H. K. (2018). Validation of the Empathy Quotient in Mainland China. *Journal of Personality Assessment*, *100*(3), 333–342. https://doi.org/10.1080/00223891.2017.1324458
